# Long-term outcomes and cost-effectiveness evaluation of robot-assisted stereotactic hematoma drainage for spontaneous intracerebral hemorrhage

**DOI:** 10.3389/fneur.2023.1291634

**Published:** 2023-11-24

**Authors:** Ke Tan, Yutao Peng, Jinping Li, Chang Liu, Libo Tao

**Affiliations:** ^1^Department of Neurosurgery, Beijing Chaoyang Hospital, Capital Medical University, Beijing, China; ^2^Center for Health Policy and Technology Evaluation, Peking University Health Science Center, Beijing, China

**Keywords:** spontaneous intracerebral hemorrhage, robotics, stereotactic neurosurgery, minimally invasive surgery, health economic evaluation

## Abstract

**Background:**

To investigate the long-term follow-up and economic estimation outcomes of hematoma drainage for spontaneous intracerebral hemorrhage (SICH) with the assistance of neurosurgical navigation and positioning planning system (referred to as robot).

**Methods:**

Data were retrospectively obtained from consecutive patients with SICH who were admitted to our single-center between March 2019 and March 2022. Different minimally invasive surgery (MIS) procedures were performed according to the inclusion/exclusion criteria. The different groups were sampled and matched using the propensity score method, with age, sex, history of stroke, hypertension, bleeding volume and site of bleeding as matching variables, and matched with inverse probability weighting using R statistical analysis software. From the time of discharge up until 1 year after the surgery, records were gathered on clinical results and medical expenditures. An analysis was conducted to compare the costs and health outcomes of both robot-assisted stereotactic hematoma drainage and neuro-endoscopic surgery, considering both short-term and long-term effects. Health outputs were measured using modified Rankin scale (mRS) and quality adjusted life years (QALYs).

**Results:**

Of the 142 patients, there were 77 patients in the robotic surgery group and 65 patients in the neuro-endoscopic surgery group. Propensity score sampling was matched, resulting in a balanced and comparable group of 37 patients in each, with the robotic surgery group [mean age (57.29 ± 12.74) years, 27 males (72.97%), hematoma volume (44.54 ± 10.49 ml), 22 deep location (59.46%)] and the neuro-endoscopic surgery group [mean age (57.27 ± 11.12) years, 27 males (72.97%), hematoma volume (44.70 ± 10.86 ml), 23 deep location (62.16%)]. At both three-month and one-year postoperative follow-up, the proportion of mRS scores ≤3 was higher in the robotic surgery group (45.95%,70.27%) than in the neuro-endoscopic surgery group (35.14%, 62.16%), but there was no statistically significant difference (*P* = 0.344, 0.461). One year after surgery, the robotic group demonstrated cost savings of ¥36,862.14 per individual and a gain of 0.062 QALYs compared to the neuro-endoscopic group.

**Conclusion:**

Our calculations based on a model for SICH suggest that robotic-assisted stereotactic drainage offers health economic benefits due to its lower cost and higher effectiveness. However, to confirm these findings, more data from multicenter, prospective randomized controlled trials with larger sample sizes are needed.

## 1 Introduction

Spontaneous intracerebral hemorrhage (SICH) is non-traumatic hemorrhage of the brain parenchyma, and its incidence is higher in China (51.8/100,000 person-years) compared with the Western countries (1). The stereotactic aspiration hematoma removal for SICH is widely applied in China, and it is included in domestic multidisciplinary guidelines (class IIa recommendation, level A evidence) (2). The MISTIE III trial, a global multi-center randomized controlled trial (RCT), found that after catheter aspiration combined with recombinant tissue plasminogen activator (rt-PA), when the residual hematoma volume was≤15 ml, the prognosis of neurological function [modified Rankin scale (mRS≤3)] at 1 year after surgery was better than that of conservative medical treatment (3), while the accuracy of catheter placement significantly affected the hematoma clearance and prognosis. While robotic-assisted technique may further enhance the efficacy of stereotactic aspiration (4, 5), it is essential to indicate whether it still possesses cost-effectiveness advantages. Meanwhile, the efficacy evaluation of minimally invasive surgery (MIS), including catheter aspiration and neuro-endoscopic hematoma removal, has been widely reported (6–9). while the comparative analysis of health economics among various MIS approaches has received less attention in the literature (10, 11). The present study evaluated the comparative analysis of efficacy, safety and health economics of robot-assisted stereotactic hematoma drainage and neuro-endoscopic surgery for the treatment of SICH based on our single-center data to provide evidence for the treatment of SICH.

## 2 Methods

### 2.1 Clinical data

This research was conducted on consecutive cases with SICH who were hospitalized for surgical treatment at the Department of Neurosurgery, Beijing Chaoyang Hospital, Capital Medical University (Beijing, China) from March 2019 to March 2022.

The inclusion criteria were as follows: (1) Supratentorial intracerebral hemorrhage ≥ 20 ml; (2) Impaired consciousness GCS ≤ 12 or limb muscle strength ≤ grade 2 on the hemiplegic side; (3) Inclusion in the emergency stroke green channel, performing cerebrovascular computed tomographic angiography (CTA) examination; (4) No significant coagulation disorders; (5) Signing informed consent form by the patient or his/her authorized family members.

**Figure 1:**
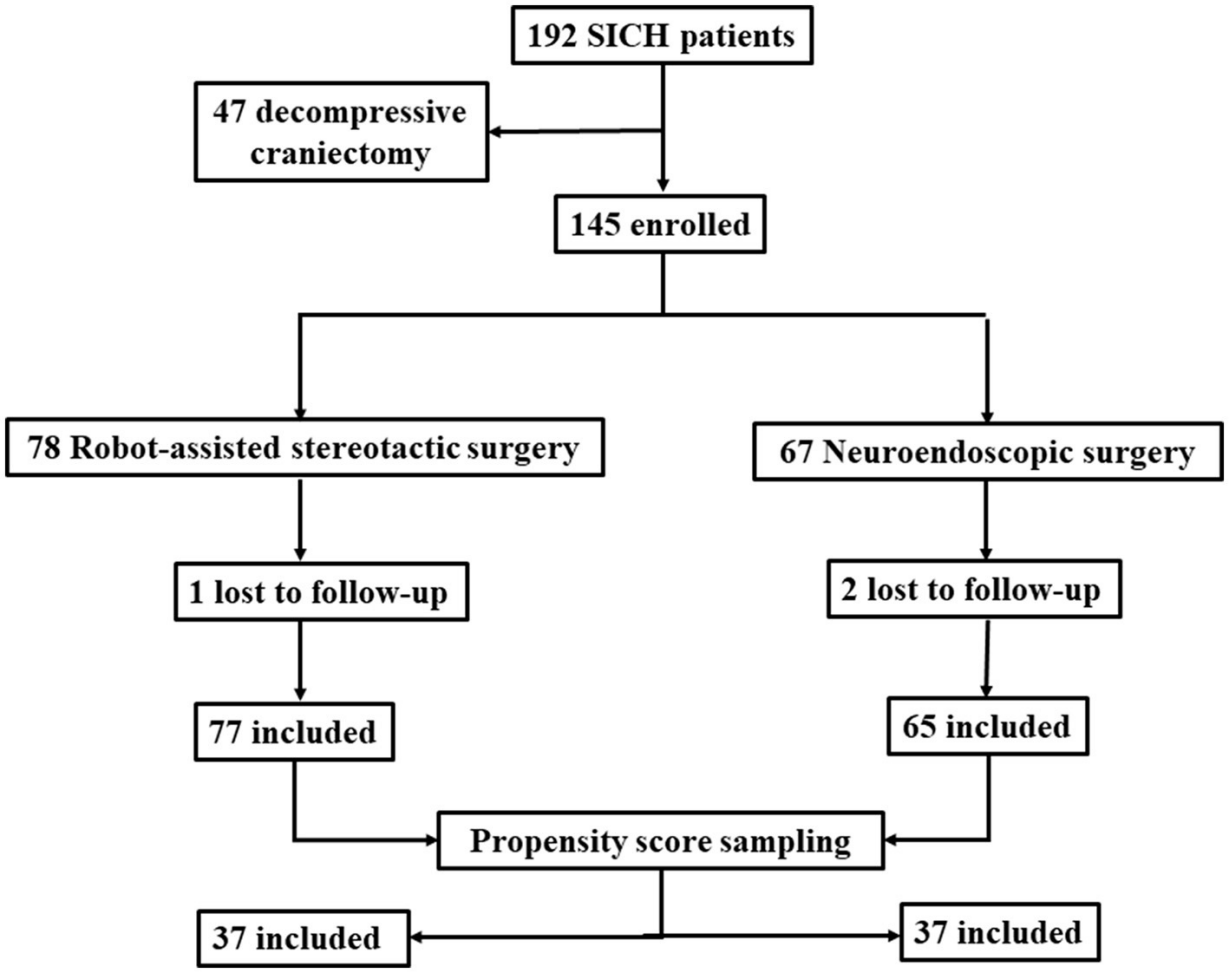
Patients' selection process.

The exclusion criteria were as follows: (1) Intracranial aneurysm, cerebrovascular malformation, brain tumor stroke as a secondary cause of hemorrhage; traumatic intracerebral hematoma; (2) Brain herniation requiring decompression surgery; (3) Brain stem hemorrhage; (4) Lack of 3 months or 1 year follow-up data. This study was approved by the Ethics Committee of Beijing Chaoyang Hospital, Capital Medical University (Approval No. 2022-ke-598). A total of 142 patients were finally included, they were assigned into 2 groups, and patients' selection process is shown in Figure 1.

### 2.2 Surgical technique

#### 2.2.1 Robot-assisted stereotactic hematoma drainage (Remebot surgery)

The scalp markers (MK-06A; Remebot, Beijing, China) were attached to the patient's forehead. The thin-slice CT scan ranged from skull base to the top of the head, and the slice thickness was 1 mm without intervals. The CTA and CT imaging data were then transferred to the robot navigation workstation (RM-100; Remebot) to perform image fusion, and a three-dimensional (3D) model was generated.

A surgical strategy was devised using a typical case of putaminal hemorrhage as a reference. The intended surgical target point was identified as the posterior section of the hematoma, located ~2 mm in proximity to the posterior limb of the internal capsule. The entry point of trajectory was in front of the coronal suture, so that the trajectory path lied within the longitudinal axis of the hematoma anteriorly and posteriorly, taking care to avoid the vessels in the cerebral sulcus. The surgical route for lobar hemorrhage was utilized to avoid the functional domains and proximity to the cortex.

The surgical procedure was performed under lidocaine local anesthesia with dexmedetomidine sedation (4 μg/ml, 20–40 μg/h continuous intravenous pumping). A three-point head frame was fixed and the skin incision was identified under the guidance of robotic arm. The skull was not exposed separately after a <10 mm length scalp dissection and it was drilled with a 5 mm drill. The dura was punctured with a monopolar electrocoagulation needle and a 12F catheter (DE-305; Sophysa, Orsay, France) was slowly placed to the target site. Cranial drilling and catheter placement was performed under the guidance of the rigid structure of a robotic arm, as opposed to free-hand manipulation under conventional navigational guidance.

A 20 ml syringe was used to aspirate until encountering initial resistance, and closely monitored for signs of active bleeding. The robotic arm was removed at the end of the procedure and the catheter was secured with scalp sutures. The follow-up CT data were reviewed within 24 h after surgery, and the thrombolytic agent urokinase was administered via intraclot injection at a dosage of 2–3×10^4^ IU, once or twice per day. The drainage catheter was typically removed within 72 h, and a follow-up CT scan was conducted to determine the hematoma volume at the end of treatment (EOT).

**Figure 2:**
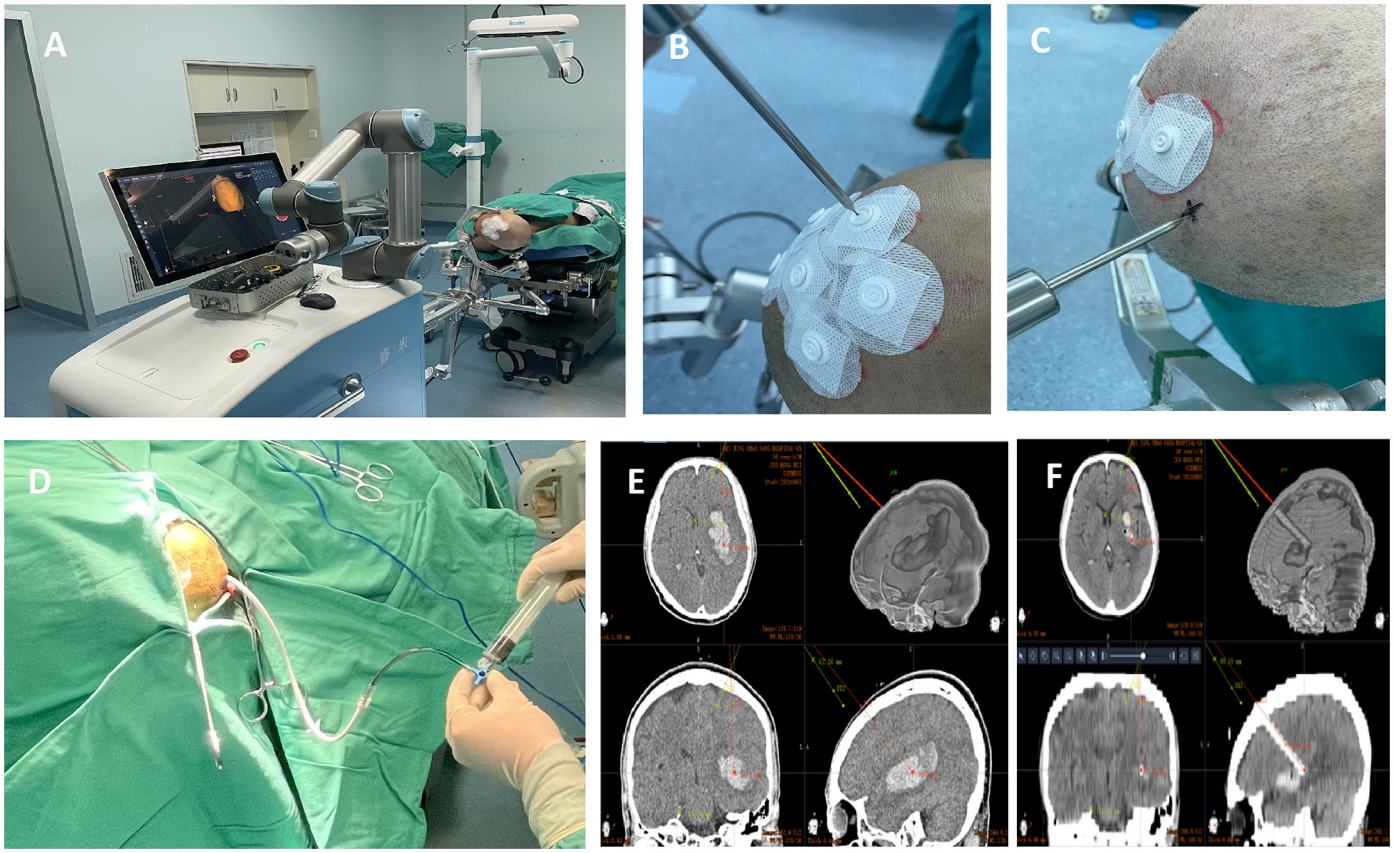
Surgical procedure of Robot-assisted stereotactic hematoma drainage (Remebot surgery). **(A)** Positioning of Remebot surgery; **(B)** Registration and verification by the scalp markers; **(C)** Marking entry points guided by robotic arms; **(D)** Drainage via soft catheter; **(E)** The entry point and target point of trajectory(red) using a trans-frontal approach; **(F)** Postoperative verification of catheter placement accuracy.

Surgical procedure is shown in Figure 2.

#### 2.2.2 Neuro-endoscopic surgery

An example of a typical putaminal hemorrhage was used for the procedure, which was carried out under general anesthesia. The surgical site was accessed by making an incision on the forehead and retracting the scalp. A circular bone flap measuring around 3 cm in diameter was created after drilling and milling the exposed skull. Next, the dura was cut and a transparent sheath was introduced to establish an endoscopic working channel. The KARL STORZ IMAGE1S Neuro-Endoscopes surgical operation system were utilized for this purpose. At the end of the procedure, depending on the clearance of the hematoma and whether it would enter the ventricle, a drainage was retained if needed. The dura was sutured and the bone flap was returned. Lobar hemorrhage was carried out on avoiding the functional domains and proximity to the cortex.

#### 2.2.3 Selection of surgical method

This study did not include patients who had experienced rapidly progressing brain herniation and had large onset hematoma volume and had undergone decompressive craniectomy. Neuro-endoscopic surgeries were suggested to patients with hemorrhage volume more than 30 ml or with early hematoma enlargement. SICH stability was required prior to Remebot surgery. All of the Remebot surgery procedures were performed by the first author. All of the Neuro-endoscopic surgical operators were well trained prior to this study. The operator's expertise and the family's preferences were also taken into account while selecting this option.

### 2.3 Outcome measurement and follow-up

The baseline characteristics, surgical complications, short-term and long-term functional outcomes, such as activities of daily living (ADL) and mRS scores at discharge, mRS scores at 3 months and 1 year after follow-up, total hospital cost, surgery-related cost, length of stay in intensive care unit (ICU), and ventilator use were assessed. Patients were followed up by outpatient clinic, telephone or online. The baseline characteristics are presented in Table 1.

**Table 1:** Characteristics of patients at baseline.

	**Remebot surgery**	**Neuro-endoscopic surgery**	***t*/χ^2^**	***P***
	***N* = 37**	***N* = 37**		
Age (years), Mean (SD)	57.29 (12.74)	57.27 (11.12)	−0.0097	0.992
Sex (Male)	27 (72.97%)	27 (72.97%)	0.000	1.000
Comorbidities				
Stroke	20 (54.05%)	20 (54.05%)	0.000	1.000
Hypertension	33 (89.19%)	32 (86.49%)	0.127	0.722
Coronary heart disease	7 (18.92%)	4 (10.81%)	0.961	0.327
Diabetes	9 (24.32%)	11 (29.73%)	0.274	0.601
Antiplatelets	7 (18.92%)	6 (16.22%)	0.093	0.760
Anticoagulant	3 (8.11%)	1 (2.70%)	1.057	0.304
Premorbid mRS			2.827	0.419
0	29 (78.38%)	30 (81.08%)		
1	4 (10.81%)	3 (8.11%)		
2	0 (0.00%)	2 (5.41%)		
3	0 (0.00%)	0 (0.00%)		
4	4 (10.81%)	2 (5.41%)		
GCS			2.906	0.234
14–15	5 (13.51%)	2 (5.41%)		
5–13	26 (70.27%)	32 (86.49%)		
3–4	6 (16.22%)	3 (8.11%)		
Mean (SD)	8.92 (0.61)	8.86 (0.47)	−0.070	0.944
Hematoma volume			0.000	1.000
≥60 ml	5 (13.51%)	5 (13.51%)		
30~59 ml	30 (81.08%)	30 (81.08%)		
20~29 ml	2 (5.41%)	2 (5.41%)		
Mean (SD)	44.54 (10.49)	44.70 (10.86)	0.065	0.948
Hematoma location			0.057	0.812
Deep	22 (59.46%)	23 (62.16%)		
Lobar	15 (40.54%)	14 (37.84%)		
Hematoma expansion	0 (0%)	6 (16.22%)	6.529	0.011
PSM score	0.53	0.47		

mRS, modified Rankin scale; GCS, Glasgow coma scale; PSM, Propensity Score Matching.

### 2.4 Statistical analysis

For the real-world data applied, the research first matched the study and control groups using a propensity score method for sampling, with age, sex, history of stroke, hypertension, bleeding volume, and bleeding site as matching variables, and inverse probability weighted matching using R statistical analysis software, resulting in the identification of 37 patients in each of the two groups that were balanced and comparable. Of the 74 patients, there were 37 patients in the robotic surgery group [mean age (57.29 ± 12.74) years, 27 males (72.97%), hematoma volume (44.54 ± 10.49 ml), 22 deep location (59.46%)] and 37 patients in the neuro-endoscopic surgery group [mean age (57.27 ± 11.12) years, 27 males (72.97%), hematoma volume (44.70 ± 10.86 ml), 23 deep location (62.16%)]. The real-world data used in descriptive statistics and economic evaluation were all the matched data of the two patient groups.

For descriptive statistics, data were statistically analyzed using Stata MP16.0 software. Sample size can be deemed as large, measurement data was expressed as mean ± standard deviation (Mean ± SD), and analysis of independent samples *t* test was used for comparison between groups; and counting information was expressed as rate (%), and χ2 test was used. Differences were considered statistically significant at *P* < 0.05.

### 2.5 Health economic evaluation

The surgical option for the study group was robot-assisted stereotactic hematoma drainage (Remebot surgery), and it was neuro-endoscopic surgery for the control group. The research utilized a Markov model to analyze intracerebral hemorrhage with a one-year state shift cycle. The simulation examined disease progression over a period of 10 years following treatment with two procedures as a long term observation. To calculate the short- and long-term costs and health outcomes [measured by quality-adjusted life years (QALYs)] of both treatments, the study adopted a societal perspective.

The model's parameter values were obtained from various sources, including real-world treatment data gathered by the Department of Neurosurgery at Beijing Chaoyang Hospital, Capital Medical University, published research literature, and consultations with clinical experts. Care was taken to ensure that all sources were properly cited and acknowledged. All cost and price data were sorted from real-world treatment data and converted to 2022 levels based on China's health care cost price index using 5% discount rate as recommended by China Guidelines for Pharmacoeconomic Evaluations and one times China's GDP per capita in 2022 (¥85,698) as the judgment threshold for the incremental cost-effectiveness ratio (ICER).

In the study, cost-effectiveness analysis results were performed with sensitivity being taken into account. The analysis concentrated on adjusting the cost, utility, and transfer probability indicators by 20%, 10%, and 10% respectively by convention, both upwards and downwards from the initial values, in order to assess any changes in the evaluation results. The baseline result was dominant, which means ICER value cannot represent the economic value precisely, therefore, incremental net benefit was conducted for showing how the economic value changes in one-way sensitivity analysis. Incremental net benefit was calculated as: ΔQALY×willingness-to-pay thresholds (China's GDP per capita in 2022)-ΔCost. A Monte Carlo simulation was used in the probabilistic sensitivity analysis to sample the cost data (using a Gamma distribution with the mean of the distribution taken as the current parameter and the standard deviation of the distribution using the standard deviation of real-world case data), the transfer probability data, and the utility data (using a Beta distribution with the mean of the distribution taken as the current parameter and the standard deviation of the distribution using a 95% confidence interval divided by 3.92 (2×1.96) as the standard deviation estimate) that were sampled 5,000 times for analysis to verify the robustness of the results.

## 3 Results

### 3.1 Surgical and follow-up results

The results of the Remebot surgery at discharge were superior to Neuro-endoscopic surgery in terms of time of ventilator use and medical cost, as shown in Table 2. Robotic surgery significantly reduced the operating time, with average time of 0.79 hours. The average length of hospital stay was 16.46 days, and the mean cost of hospitalization was ¥ 50,516. There was no significant difference in mRS and ADL at discharge between the two groups.

**Table 2:** Surgical and follow-up results.

	**Remebot surgery; *N* = 37**	**Neuro-endoscopic surgery; *N* = 37**	**t/χ^2^**	***P***
Time to surgery (h)	40.38 (24.51)	9.62 (9.55)	−7.111	0.000
Time of surgery (h)	0.79 (0.39)	2.72 (0.66)	15.356	0.000
EOT (End of treatment) Remain ICH volume			1.057	0.304
< 15 ml	36 (97.30%)	34 (91.89%)		
≥15 ml	1 (2.70%)	3 (8.11%)		
Median (Quartiles)	5.22 (3.13)	5.38 (5.09)	0.1651	0.869
Complications				
Rebleeding	1 (2.70%)	2 (5.41%)	0.347	0.556
Intracranial infection	0 (0.00%)	3 (8.11%)	3.127	0.077
ADL at discharge	25.81 (30.20)	26.35 (26.26)	0.082	0.935
mRS score at discharge			5.881	0.318
0	0 (0%)	0 (0%)		
1	2 (2.6%)	0 (0%)		
2	0 (0%)	1 (4.6%)		
3	1 (2.6%)	2 (7.7%)		
4	11 (39.0%)	13 (35.4%)		
5	23 (55.8%)	19 (49.2%)		
6	0 (0%)	2 (3.1%)^*^		
mRS score at 3 months			0.897	0.344
0~3	17 (45.95%)	13 (35.14%)		
4~6	20 (54.05%)	24 (64.86%)		
mRS score at 1 year			0.544	0.461
0~3	26 (70.27%)	23 (62.16%)		
4~6	11 (29.73%)	14 (37.84%)		
Total hospital cost (¥) Mean (SD)	50,516 (29,806)	89,155 (42,647)	4.517	0.000
Total hospital cost (¥); Median(Quartiles)	38,377 (30,597, 59,161)	74,674 (54,500, 119,924)	4.517	0.000
Surgery-related cost (¥)	11,663 (247)	29,669 (10,462)	10.463	0.000
Length of hospital stay; Mean (SD)	16.46(8.43)	16.08(5.73)	−0.226	0.822
Length of hospital stay; Median (Quartiles)	14 (12,20)	14 (12,19)	−0.226	0.822
Length of ICU stay (d)	4.38 (6.11)	7.30 (6.66)	1.964	0.053
Time of ventilator use (h)	17.65 (49.35)	79.92 (94.14)	3.563	0.001

EOT, End of treatment; ADL, activity of daily living.

* mRS 6 coverage of disease specific mortality only.

**Figure 3:**
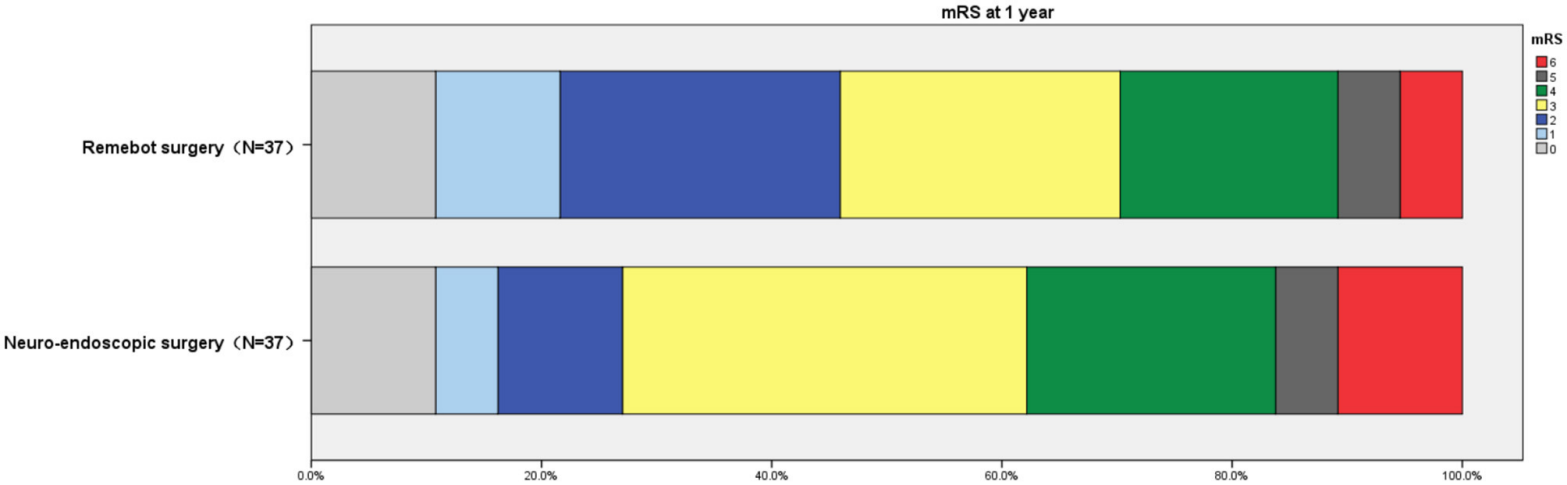
The proportion of mRS scores at one-year follow-up.

The results obtained at 3 months and 1 year follow-up showed a higher proportion of mRS scores ≤ 3 in the Remebot surgery group (45.95%,70.27%) vs. those in the Neuro-endoscopic surgery group (35.14%, 62.16%), but there was no statistically significant difference (*P* = 0.344, 0.461). Figure 3 shows the proportion of mRS scores in each group at one-year follow-up.

The incidence of complications of Remebot surgery was relatively low. Of all 77 cases, there were two cases of asymptomatic rebleeding at the drainage channel and one case of intracranial infection, one case of postoperative residual hematoma volume ≥15 ml.

### 3.2 Results of the health economics evaluation

#### 3.2.1 Model's structure and values of parameters

**Figure 4:**
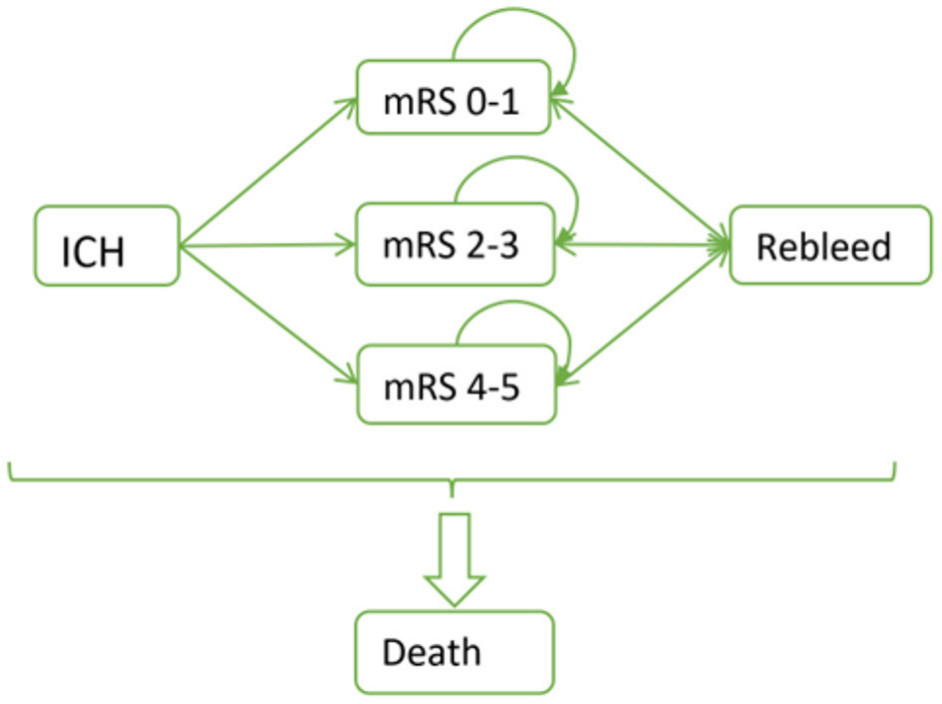
The Markov model of ICH. ICH, intracerebral hemorrhage. mRS, modified Rankin scale.

The Markov model used for the health economics evaluation is illustrated in Figure 4. Patients are subjected to surgical procedures and are categorized based on the mRS scale, ranging from 0–1, 2–3, 4–5 or mortality. These categories are determined by evaluating the real-world outcome data. After the surgery, there is a likelihood of rebleeding in each subsequent year. Depending on the severity, patients may either return to their initial state, experience progression toward a more severe state or succumb to death.

The cost parameters of our model take a social perspective, considering the complete perioperative expense of a procedure. This includes the medical care cost and the patient's lost wages calculated using the average number of days spent in the hospital. Main medical care costs, which were over all hospitalization cost of each group, were calculated according to real-world clinical records based on the same cohort data from Department of Neurosurgery, Beijing Chaoyang Hospital, the Capital Medical University from March 2019 to March 2022. Total hospital costs include itemized fees for examination, drugs, medical services and hospitalization. Surgery-related costs include surgical fees, anesthesia and surgical consumables. This study calculates patients' expenditure on item-based payment from the perspective of the community, not from the perspective of the hospital, so we do not touch on issues such as capital costs and depreciation of equipment. To avoid outlier effects, for key parameters such as total hospital cost and length of hospital stay, median values from real-world data were adopted. Additionally, we have accounted for the rehabilitation expenses for ICH patients as reported by Yu (12). The regular rehabilitation training and maintenance treatment costs are considered for patients with postoperative mRS scores of 2–3 and 4–5. Furthermore, the total perioperative cost of another surgery according to real-world data was calculated for patients with rebleeding. The distribution of mRS scores in two groups at the one-year follow-up was obtained through real-world data analysis to determine health output parameters. According to the data, Markov model inter-state transfer probabilities were calculated. Subsequent costs, the values for patients' re-bleeding rates, subsequent re-bleeding rates, disease-specific mortality and utility values for each health state were derived from previously published studies (13, 14). The values for specific parameters are presented in Table 3.

**Table 3:** The sources and settings of model parameters.

**Projects**	**Values**	**Sources**
**Cost (**¥**)**		
Remebot surgery	38,377.00	Real-world data
Neuro-endoscopic surgery	74,674.00	Real-world data
mRS 2-3 maintenance treatment	15,083.87	Yu (12)
mRS 4-5 maintenance treatment	26,144.80	Yu (12)
Length of hospital stay in the Remebot surgery	14.00	Real World Data
Length of hospital stay in the Endoscopic surgery	14.00	Real-world data
Daily GDP per capita	234.79	2022 GDP per capita/365
**Transfer probability**		
**Remebot surgery post-operative distribution**		
mRS 0-1	0.2162	Real-world data
mRS 2-3	0.4865	Real-world data
mRS 4-5	0.2432	Real-world data
**Endoscopic surgery post-operative distribution**		
mRS 0-1	0.1622	Real-world data
mRS 2-3	0.4595	Real-world data
mRS 4-5	0.2703	Real-world data
Rebleeding after primary ICH	0.0208	Aviv et al. (13)
Rebleeding after secondary ICH	0.0392	Aviv et al. (13)
**Probability of death in various states**		
After Remebot surgery	0.0541	Real-world data
After Endoscopic surgery	0.1081	Real-world data
mRS 0-1	0.0241	Arora et al. (14)
mRS 2-3	0.0267	Arora et al. (14)
mRS 4-5	0.0739	Arora et al. (14)
Rebleeding	0.0470	Aviv et al. (13)
**Utility values**		
mRS 0-1	0.90	Aviv et al. (13)
mRS 2-3	0.75	Aviv et al. (13)
mRS 4-5	0.25	Aviv et al. (13)
Discount rate	5%	China Guidelines for Pharmacoeconomic Evaluations

#### 3.2.2 Results of the baseline analysis

It was estimated that 1,000 patients with SICH were initially enrolled in the study and control groups, and the model was extrapolated from 1 to 10 years after surgery to perform short- and long-term economic evaluations.

After conducting calculations, it was determined that the individuals in the study group were able to save ¥ 36,862.14 per capita and experience an increase of 0.062 QALYs compared to the control group, following 1 year of surgical intervention for SICH patients. Additionally, after a period of 10 years, the study group was found to have saved ¥ 40,442.19 per capita and gained 0.463 QALYs more than the control group. The threshold was set as one times China's GDP per capita in 2022 (¥ 85,698) per QALY, therefore health gain was ¥ 39,678. Incremental net benefit of Remebot surgery compared to control group was ¥80,120. The results of both short- and long-term health economics evaluations indicated that the study group had an absolute economic advantage over the control group in terms of a lower cost and a greater efficacy. The results of the baseline analysis are presented in Table 4.

**Table 4:** The results of the cost-utility analysis.

	**Costs per capita(¥)**	**QALYs**	**ΔC(¥)**	**ΔQALY**	**ICER(¥/QALY)**
**Short-term(1year)**					
Endoscopic surgery	91,614.16	0.549	−36,862.14	0.062	−594,550.64 (Dominant)
Remebot surgery	54,752.02	0.611			
**Long-term(10 years)**					
Endoscopic surgery	127,144.33	3.836	−40,442.19	0.463	−87,348.14 (Dominant)
Remebot surgery	86,702.15	4.299			

#### 3.2.3 Results of the sensitivity analysis

**Figure 5:**
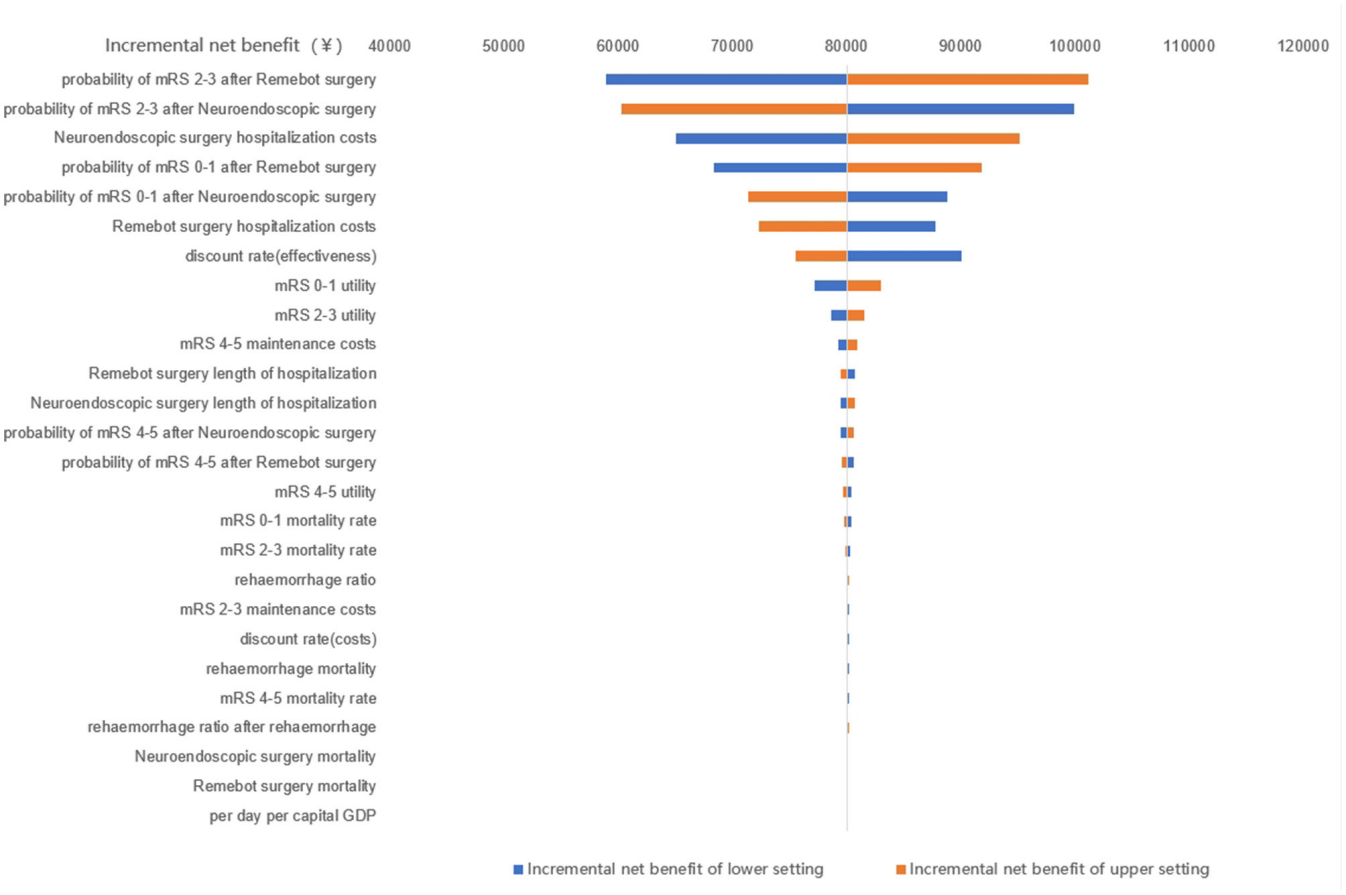
The Tornado diagram of the single-factor sensitivity analysis. mRS, modified Rankin scale.

After conducting the single-factor sensitivity analysis, it was revealed that altering individual parameter values had an impact on outcomes. The results indicated that two specific parameter categories, namely the distribution of postoperative mRS scores and overall hospital costs, significantly contributed to the observed outcomes. To be specific, the incremental net benefit was ¥ 80,120 as baseline result shown, while the distribution of postoperative mRS scores of Remebot surgery increased by 10%, incremental net benefit grew into ¥ 101,222. while the distribution of postoperative mRS scores of Remebot surgery decreased by 10%, incremental net benefit dropped into ¥ 58,948; while the overall cost of Remebot surgery increased by 20%, incremental net benefit dropped into ¥ 72,337. while the overall cost of Remebot surgery decreased by 20%, incremental net benefit grew into ¥ 87,832. The Tornado diagram is shown in Figure 5.

**Figure 6:**
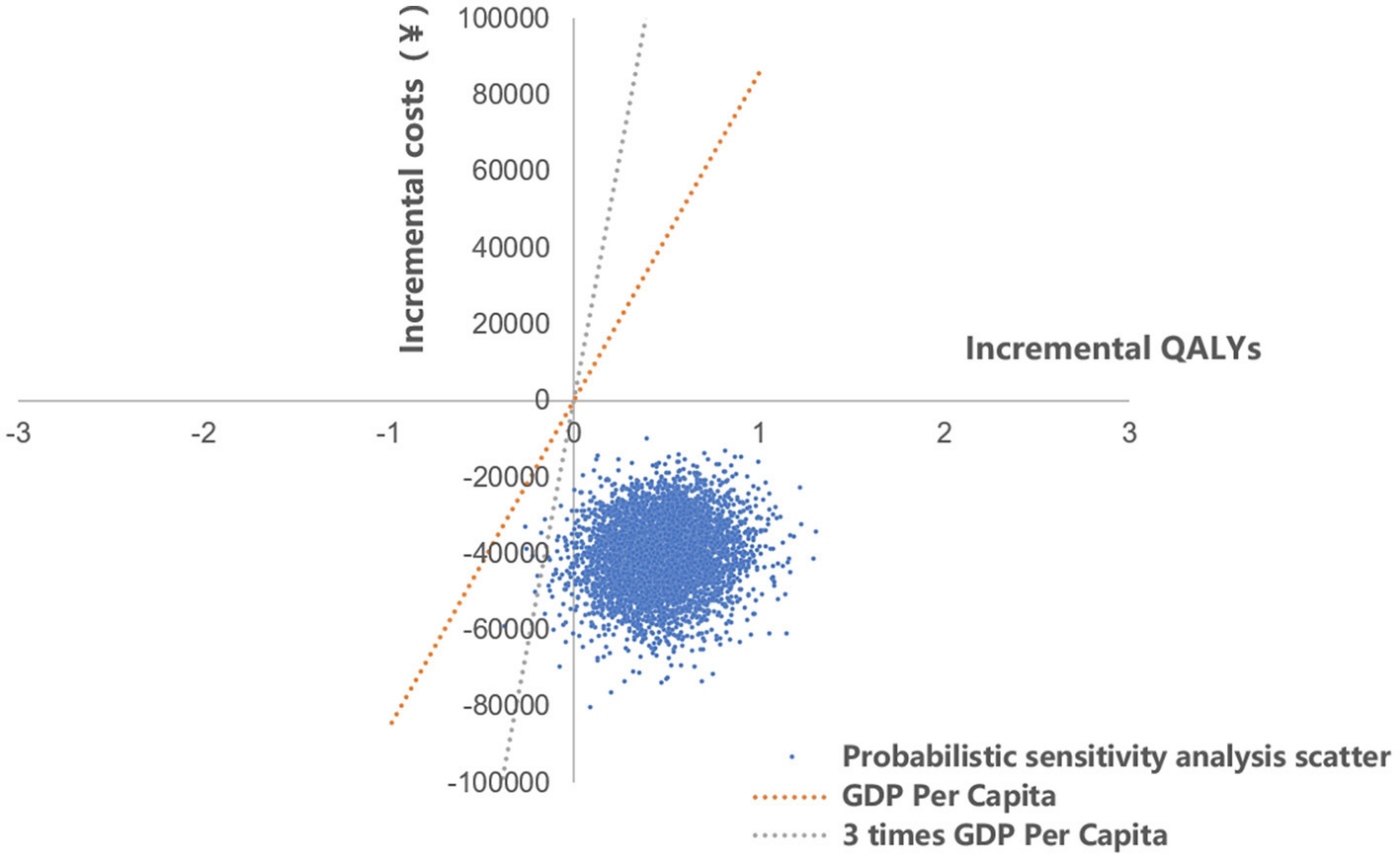
The scatterplot of probabilistic sensitivity analysis. QALYs, quality adjusted life years.

**Figure 7:**
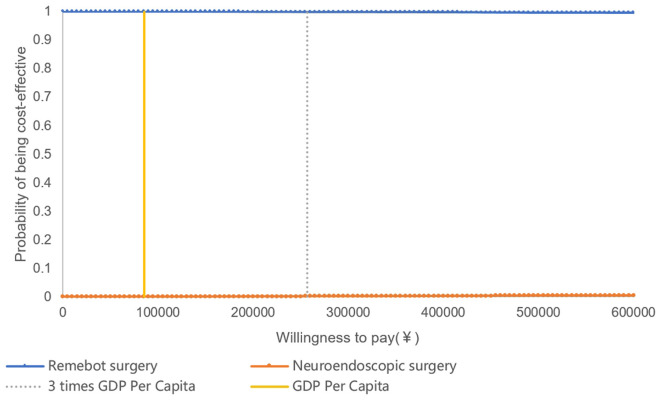
The cost-effective acceptability curve. QALYs, quality adjusted life years.

The results of probabilistic sensitivity analysis, which examined the impact of varying multiple parameter values simultaneously, revealed that the majority of sample calculations fell below the threshold line. This suggested that, in the majority of cases, the study group was more cost-effective compared with the control group. The curve of cost-effectiveness acceptability proposed that the economic feasibility of the study group was assured with a 100% likelihood when the willingness to pay was equal to one time the GDP per capita. This finding underscores the reliability and resilience of the outcomes of the health economics evaluation (Figures 6, 7).

## 4 Discussion

SICH is characterized by a high rate of disability, and the MIS technique becomes part of the standardized treatment of SICH for improving outcomes by removing hematoma, while minimizing surgical invasion. China is a vast country with varying levels of medical technology, and the surgical option for SICH varies in different regions. We are a regional neurosurgical center located in the eastern part of Beijing. Our facility serves as a community hospital for a population of 3,400,000 residents, and delivers tertiary acute stroke care to a larger population of 4,370,000 individuals. MIS techniques, including stereotactic surgery, neuro-endoscopic surgery, conventional microscopic surgery, and bedside CT-guided free-hand catheter surgery have all been used to treat SICH. Our single-center data are therefore representative of the current state of local SICH treatment in China to some extent.

The MISTIE III trial found that catheter drainage combined with rt-PA treatment improved the neurological prognosis of SICH when the residual hematoma volume was ≤15 ml (3). A multicenter RCT conducted in China also compared stereotactic drainage combined with urokinase with small bone window craniotomy for microscopic hematoma removal, improving the patient's ability to perform daily activities at 90 days (15). Our robot-assisted stereotactic drainage technique has some similarities and differences with the requirements of the MISTIE III trial's 10-step protocol (3). (1) We used a trans-frontal approach in our procedure for a putaminal hemorrhage. Placing the soft catheter at the target location was crucial in the MISTIE III trial. We decided to place the target more posteriorly near the internal capsule to decompress the corticospinal tract as much as possible. (2) The use of a robotic arm for guiding placement was found to be more accurate than conventional navigation (4, 5). (3) To minimize the risk of CSF loss and shift, as well as postoperative CSF leakage and infection, we opted for a less invasive method. Instead of exposing the skull alone to drill and cut the dura, we used a 5 mm drill followed by electro-coagulation of the dura. (4) Our procedure can be performed under local anesthesia with dexmedetomidine sedation, eliminating the need for general anesthesia with tracheal intubation. This approach reduces postoperative ventilator support and ICU stay, which is particularly beneficial for elderly patients and lowers medical costs (16). The present study confirmed the feasibility and safety of performing SICH drainage under local anesthesia with dexmedetomidine sedation.

Regarding time to surgery, similar to the MISTIE III trial (17), we do not pursue ultra-early surgery. Stereotactic drainage is limited for patients with SICH who may experience early hematoma expansion or instability within 6 h of onset. As a result, neuro-endoscopic techniques may be more appropriate for this group of patients. Waiting for the hematoma to stabilize before performing drainage surgery can significantly reduce the incidence of rebleeding. The present study also showed that performing surgery more than 24 h and <72 h after SICH onset could lead to neurological improvement (18). The volume of residual hematoma at the end of treatment (EOT) should be given greater consideration, as it significantly impacts the neurological outlook for SICH patients (19). Urokinase has a concentration gradient that spreads within the clot, and an appropriate catheter placement is an important factor in reducing the EOT volume that can attenuate the risk of infection associated with excessive use of urokinase and prolonged drainage, exemplifying the importance of using robot-assisted technology to improve the accuracy of catheter placement.

Regarding surgical complications, two instances of asymptomatic rebleeding occurred in the Remebot surgery. Rebleeding is often associated with poor blood pressure control, abnormal coagulation, and puncture injury, etc. Care should be taken to avoid vessels in the sulcus on CTA when formulating the surgical plan preoperatively, and particular attention should be paid to the elderly patients with cerebral atrophy. In one case, an excessive hematoma residue was found during postoperative CT review due to catheter dislodgment, which should be documented for proper postoperative management. The incidence of infection was low in the whole group, and only one case was identified after excessive ventricular drainage prior to hospitalization.

Neuro-endoscopic surgery is in line with the trend toward MIS, and as a result of advances in navigation technology and endoscopic instrumentation, the practice of Neuro-endoscopic surgery has become increasingly prevalent in recent years. Several studies have confirmed that neuro-endoscopic surgery can achieve good immediate and long-term outcomes for SICH (20), and there have also been retrospective comparative studies reporting superior outcomes to stereotactic surgery (7). The difference between the results of these studies and the present study could be related to two factors: firstly, the stereotactic technique used was not detailed in the above-mentioned studies; robot-assisted surgery has a higher precision and should theoretically have greater surgical safety and efficacy; Surgeon training experience and technique can also affect surgical outcomes; secondly, in cases of SICH with a large clot volume, significantly elevated intracranial pressure, and hematoma expansion, it is recommended to promptly remove the clot instead of waiting for the hematoma to stabilize. This decision should be made based on careful consideration of surgical indications (21). Compared to open surgery, stereotactic drainage surgery is associated with a more apparent minimally invasive advantage for stable hematoma at small-to-medium sizes and deep sites. This is particularly true when the procedure is performed under local anesthesia, providing notable economic benefits.

A study from the University of Barcelona reported economic advantage for neuro-endoscopic surgery (60,703.89/QALY) over catheter-based surgery (76,533.13/QALY) through a quality-adjusted life year (QALY) model analysis (10). A study on the economic estimate of MIS treatment of SICH in four third-level hospitals located in Beijing, Shanghai, Hangzhou, and Suzhou reported that both the small bone window microscopic group and endoscopic group had better mRS scores at 6 months than the stereotactic group, and the stereotactic group had a definite cost-effectiveness and safety advantage (11). In the present study, based on immediate outcomes, long-term follow-up data, and the health economics evaluation model, it was revealed that robotic-assisted stereotactic surgery still had an absolute advantage in terms of cost-effectiveness, while the 3-month and 1-year mRS scores were also greater than those of the neuro-endoscopic and microscopic groups.

There are also some limitations in the present study. Firstly, although propensity scoring methods were used for sampling and matching in this retrospective study, there was still some bias in the selection of surgical indications based on hematoma stability and operator experience. The indications for robotic surgery are more limited than endoscopic surgery, including hematoma stability. Thus, further investigation is needed to clarify the effectiveness of stereotactic surgery compared to conservative medical treatment for small to medium volume SICH of 20–30 ml through a well-designed prospective multicenter RCT. Secondly, in moderate and moderate-to-large volume hematomas, the efficacy of robotic-assisted stereotactic surgery vs. other minimally invasive surgical techniques, such as neuro-endoscopic surgery, might be influenced by multiple factors, including the hematoma volume threshold, and hematoma stability, which warrant further exploration through a large-scale prospective multicenter RCT.

## Data availability statement

The raw data supporting the conclusions of this article will be made available by the authors, without undue reservation.

## Ethics statement

The studies involving humans were approved by the Ethics Committee of Beijing Chaoyang Hospital, Capital Medical University. The studies were conducted in accordance with the local legislation and institutional requirements. The participants provided their written informed consent to participate in this study.

## Author contributions

KT: Conceptualization, Formal analysis, Funding acquisition, Methodology, Project administration, Resources, Writing—original draft. YP: Data curation, Investigation, Writing—original draft. JL: Supervision, Writing—review & editing. CL: Software, Writing—original draft. LT: Methodology, Software, Supervision, Writing—review & editing.

## References

[1] Wu S Wu B Liu M Chen Z Wang W Anderson CS. Stroke in China: advances and challenges in epidemiology, prevention, and management. Lancet Neurol. (2019) 18:394–405. 10.1016/S1474-4422(18)30500-3 30878104

[2] Chinese Society of Neurosurgery Chinese Physicians Association of Emergency Chinese Society of Neurology Cerebrovascular Disease Group National Health Care Commission Stroke Screening and Prevention Project Committee. Chinese multidisciplinary diagnosis and treatment guidelines for hypertensive cerebral hemorrhage. China Emerg Med. (2020) 40:689–702. 10.3969/j.issn.1002-1949.2020.08.001

[3] Awad IA Polster SP Carrión-Penagos J Thompson RE Cao Y Stadnik A. Surgical performance determines functional outcome benefit in the minimally invasive surgery plus recombinant tissue plasminogen activator for intracerebral hemorrhage evacuation (MISTIE) procedure. Neurosurgery. (2019) 84:1157–68. 10.1093/neuros/nyz077 30891610 PMC6537634

[4] Alan N Lee P Ozpinar A Gross BA Jankowitz BT. Robotic stereotactic assistance (ROSA) utilization for minimally invasive placement of intraparenchymal hematoma and intraventricular catheters. World Neurosurg. (2017) 108:996.e997–996.e910. 10.1016/j.wneu.2017.09.027 28919568

[5] Wang T Zhao Q-J Gu J-W Shi T-J Yuan X Wang J. Neurosurgery medical robot Remebot for the treatment of 17 patients with hypertensive intracerebral hemorrhage. Int J Med Robot. (2019) 15:e2024. 10.1002/rcs.2024 31267676

[6] Kellner CP Song R Pan J. Long-term functional outcome following minimally invasive endoscopic intracerebral hemorrhage evacuation. J Neurointerv Surg. (2020) 12:489–94. 10.1136/neurintsurg-2019-015528 31915207 PMC7231458

[7] Guo W Liu H Tan Z Zhang X Gao J Zhang L. Comparison of endoscopic evacuation, stereotactic aspiration, and craniotomy for treatment of basal ganglia hemorrhage. J Neurointerv Surg. (2020) 12:55–61. 10.1136/neurintsurg-2019-014962 31300535 PMC6996102

[8] Zhao Z Xiao J Wang J Meng X Li C Xin T. Individualized CT image-guided free-hand catheter technique: a new and reliable method for minimally invasive evacuation of basal ganglia hematoma. Front Neurosci. (2022) 16:947282. 10.3389/fnins.2022.947282 36090281 PMC9461711

[9] Xiong R Li F Chen X. Robot-assisted neurosurgery versus conventional treatment for intracerebral hemorrhage: A systematic review and meta-analysis. J Clin Neurosci. (2020) 82:252–9. 10.1016/j.jocn.2020.10.045 33248949

[10] Mosteiro A Amaro S Torné R Pedrosa L Hoyos J Llull L. Minimally invasive surgery for spontaneous intracerebral hematoma. Real-life implementation model and economic estimation. Front Neurol. (2022) 13:884157. 10.3389/fneur.2022.884157 35585845 PMC9108381

[11] Hu SM Ren LH Hua LY Gong Y Hou ZY. Health economics evaluation of minimally invasive surgical treatment for spontaneous intracerebral hemorrhage - a multicenter study based on real-world data. Chin Clini Neurosci. (2022) 30:158–67.

[12] Yu J. Studies on the Effects of Community-Based Rehabilitation Therapy on the Function, Economic Analysis and Risk Factors for Stroke Patients. Shanghai Fudan University. (2008).

[13] Aviv RI Kelly AG Jahromi BS Benesch CG Young KC. The cost-utility of CT angiography and conventional angiography for people presenting with intracerebral hemorrhage. PLoS ONE. (2014) 9:e96496. 10.1371/journal.pone.0096496 24824194 PMC4019473

[14] Arora N Makino K Tilden D Lobotesis K Mitchell P Gillespie J. Cost-effectiveness of mechanical thrombectomy for acute ischemic stroke: an Australian payer perspective. J Med Econ. (2018) 21:799–809. 10.1080/13696998.2018.1474746 29741126

[15] Sun H Liu H Li D Liu L Yang J Wang W. An effective treatment for cerebral hemorrhage: minimally invasive craniopuncture combined with urokinase infusion therapy. Neurol Res. (2010) 32:371–7. 10.1179/016164110X12670144526147 20483003

[16] Bardutzky J Hieber M Roelz R Meckel S Lambeck J Niesen WD. Cerebral amyloid angiopathy-related intracerebral hemorrhage: Feasibility and safety of bedside catheter hematoma evacuation with urokinase. Clin Neurol Neurosurg. (2020) 190:105655. 10.1016/j.clineuro.2019.105655 31901893

[17] Polster SP Carrión-Penagos J Lyne SB Gregson B Cao Y Thompson R. Intracerebral hemorrhage volume reduction and timing of intervention versus functional benefit and survival in the MISTIE III and STICH trials. Neurosurgery. (2021) 88:961–70. 10.1093/neuros/nyaa572 33475732 PMC8190461

[18] Scaggiante J Zhang X Mocco J Kellner CP. Minimally invasive surgery for intracerebral hemorrhage. Stroke. (2018) 49:2612–20. 10.1161/STROKEAHA.118.020688 30355183

[19] de Havenon A Joyce E Yaghi S Ansari S Delic A Taussky P. End-of-treatment intracerebral and ventricular hemorrhage volume predicts outcome: a secondary analysis of MISTIE III. Stroke. (2020) 51:652–4. 10.1161/STROKEAHA.119.028199 31842688 PMC7000178

[20] Liu H Wu X Tan Z Guo H Bai H Wang B. Long-term effect of endoscopic evacuation for large basal ganglia hemorrhage with GCS scores ≦ 8. Front Neurol. (2020) 11:848. 10.3389/fneur.2020.00848 32922354 PMC7457040

[21] Al-Kawaz MN Li Y Thompson RE Avadhani R de Havenon A Gruber J. Intracranial pressure and cerebral perfusion pressure in large spontaneous intracranial hemorrhage and impact of minimally invasive surgery. Front Neurol. (2021) 12:729831. 10.3389/fneur.2021.729831 34512537 PMC8427275

