# Supplementary material for: Long-term outcomes and cost-effectiveness evaluation of robot-assisted stereotactic hematoma drainage for spontaneous intracerebral hemorrhage
Source: Front Neurol. 2023 Nov 24;14:1291634. doi: 10.3389/fneur.2023.1291634 (PMC10704362; doi:10.3389/fneur.2023.1291634)
Supplement: Supplementary file 1 [file Presentation_1.pdf]

Appendix

The CHEERS 2022 Checklist.

（Long-term outcomes and cost-effectiveness evaluation of robot-assisted stereotactic hematoma drainage for spontaneous intracerebral hemorrhage）

Section/topic	Item No.	Guidance for reporting	Reported in section
**Title**			
Title	1	Identify the study as an economic evaluation and specify the interventions being compared.	reported
**Abstract**			
Abstract	2	Provide a structured summary that highlights context, key methods, results, and alternative analyses.	reported
**Introduction**			
Background and objectives	3	Give the context for the study, the study question, and its practical relevance for decision making in policy or practice.	reported
**Methods**			
Health economic analysis plan	4	Indicate whether a health economic analysis plan was developed and where available.	reported
Study population	5	Describe characteristics of the study population (such as age range, demographics, socioeconomic, or clinical characteristics).	reported
Setting and location	6	Provide relevant contextual information that may influence findings.	reported
Comparators	7	Describe the interventions or strategies being compared and why chosen.	reported
Perspective	8	State the perspective(s) adopted by the study and why chosen.	reported
Time horizon	9	State the time horizon for the study and why appropriate.	reported
Discount rate	10	Report the discount rate(s) and reason chosen.	reported
Selection of outcomes	11	Describe what outcomes were used as the measure(s) of benefit(s) and harm(s).	reported
Measurement of outcomes	12	Describe how outcomes used to capture benefit(s) and harm(s) were measured.	reported
Valuation of outcomes	13	Describe the population and methods used to measure and value outcomes.	reported
Measurement and valuation of resources and costs	14	Describe how costs were valued.	reported
Currency, price date, and conversion	15	Report the dates of the estimated resource quantities and unit costs, plus the currency and year of conversion.	reported
Rationale and description of model	16	If modelling is used, describe in detail and why used. Report if the model is publicly available and where it can be accessed.	reported
Analytics and assumptions	17	Describe any methods for analysing or statistically transforming data, any extrapolation methods, and approaches for validating any model used.	reported
Characterizing heterogeneity	18	Describe any methods used for estimating how the results of the study vary for subgroups.	Not reported
Characterizing distributional effects	19	Describe how impacts are distributed across different individuals or adjustments made to reflect priority populations.	reported
Characterizing uncertainty	20	Describe methods to characterise any sources of uncertainty in the analysis.	reported

Approach to engagement with patients and others affected by the study	21	Describe any approaches to engage patients or service recipients, the general public, communities, or stakeholders (such as clinicians or payers) in the design of the study.	reported
**Results**			
Study parameters	22	Report all analytic inputs (such as values, ranges, references) including uncertainty or distributional assumptions.	reported
Summary of main results	23	Report the mean values for the main categories of costs and outcomes of interest and summarise them in the most appropriate overall measure.	reported
Effect of uncertainty	24	Describe how uncertainty about analytic judgments, inputs, or projections affect findings. Report the effect of choice of discount rate and time horizon, if applicable.	reported
Effect of engagement with patients and others affected by the study	25	Report on any difference patient/service recipient, general public, community, or stakeholder involvement made to the approach or findings of the study	reported
**Discussion**			
Study findings, limitations, generalizability, and current knowledge	26	Report key findings, limitations, ethical or equity considerations not captured, and how these could affect patients, policy, or practice.	reported
**Other relevant information**			
Source of funding	27	Describe how the study was funded and any role of the funder in the identification, design, conduct, and reporting of the analysis	reported
Conflicts of interest	28	Report authors conflicts of interest according to journal or International Committee of Medical Journal Editors requirements.	reported
